# Estimates of marker effects for measures of milk flow in the Italian brown Swiss dairy cattle population

**DOI:** 10.1186/1746-6148-8-199

**Published:** 2012-10-23

**Authors:** Kent A Gray, Christian Maltecca, Alessandro Bagnato, Marlies Dolezal, Attilio Rossoni, Antonia B Samore, Joseph P Cassady

**Affiliations:** 1 Animal Breeding and Genetics, Department of Animal Science, North Carolina State University, Raleigh, NC, USA; 2 Department of Health, Animal Science and Food Safety, Universitá degli Studi di Milano, Milano, Italy; 3 Institut für Populationsgenetik, University of Veterinary Medicine, Vienna, Austria; 4 Italian Brown Breeders Association, Bussolengo, Italy

**Keywords:** Milk flow, GWAS, Milkability

## Abstract

**Background:**

Milkability is a complex trait that is characterized by milk flow traits including average milk flow rate, maximum milk flow rate and total milking time. Milkability has long been recognized as an economically important trait that can be improved through selection. By improving milkability, management costs of milking decrease through reduced labor and improved efficiency of the automatic milking system, which has been identified as an important factor affecting net profit. The objective of this study was to identify markers associated with electronically measured milk flow traits, in the Italian Brown Swiss population that could potentially improve selection based on genomic predictions.

**Results:**

Sires (n = 1351) of cows with milk flow information were genotyped for 33,074 single nucleotide polymorphism (SNP) markers distributed across 29 *Bos taurus* autosomes (BTA). Among the six milk flow traits collected, ascending time, time of plateau, descending time, total milking time, maximum milk flow and average milk flow, there were 6,929 (time of plateau) to 14,585 (maximum milk flow) significant SNP markers identified for each trait across all BTA. Unique regions were found for each of the 6 traits providing evidence that each individual milk flow trait offers distinct genetic information about milk flow. This study was also successful in identifying functional processes and genes associated with SNPs that influences milk flow.

**Conclusions:**

In addition to verifying the presence of previously identified milking speed quantitative trait loci (QTL) within the Italian Brown Swiss population, this study revealed a number of genomic regions associated with milk flow traits that have never been reported as milking speed QTL. While several of these regions were not associated with a known gene or QTL, a number of regions were associated with QTL that have been formerly reported as regions associated with somatic cell count, somatic cell score and udder morphometrics. This provides further evidence of the complexity of milk flow traits and the underlying relationship it has with other economically important traits for dairy cattle. Improved understanding of the overall milking pattern will aid in identification of cows with lower management costs and improved udder health.

## Background

In dairy cattle, traits influencing efficiency of production can be characterized as production or functional traits. Traits classified as functional, increase efficiency by reducing costs of inputs. Milkability, health, fertility, feed efficiency and calving ease all belong to this group of functional, cost-saving traits [1].

Milkability is a complex trait that is most often characterized by average milk flow rate (AVGF), maximum milk flow rate (MMF) and total milking time (TMT) [2,3].

Milkability has long been recognized as an economically important trait that can be improved through selection [4,5]. By improving milkability, management costs of milking decrease through reduced labor and improved efficiency of the automatic milking system [1], which has been identified as an important factor affecting net profit [6].

In 2005, the Italian Brown Breeders Association (ANARB) made an update to their selection index (TMI) to include milking speed measured as AVGF. However, milking speed is unfavorably correlated with other economically important traits, particularly somatic cell score (SCS, r=0.46) [7]. Therefore with the inclusion of milking speed in the selection index, SCS was also included with a slight negative weight in the TMI. This negative weight was introduced in the TMI to offset the predicted consequence that an increase in milking speed could potentially increase SCS resulting in a possible increase of mastitis [7].

Gray et al. [3] showed that a combination of milk flow parameters describing the overall patterns of milk removal would be an advantageous selection strategy for improved milkability, when compared to use of a single variable such as AVGF. Traits previously shown to be unfavorably correlated [8] with milking speed (SCS, udder score) could respond favorably to selection by including both milking flow and milking time measures in a sub-index with appropriate weights [3].

By exploiting the advances in molecular genetics and bioinformatics tools an increased understanding of milk flow traits on a molecular level could be achieved. Development of dense SNP marker panels has provided an opportunity to perform genome wide association studies (GWAS) to determine biological differences that exist in an animal’s genetic makeup [9].

Molecular genetic markers that are associated with phenotypic variation of complex traits, provide information that may be utilized through marker assisted selection [10] or genomic selection [11]. Using information available from markers in selection can increase the accuracy and efficiency of a breeding program when compared to traditional breeding schemes [12].

Only a few quantitative trait loci (QTL) have previously been associated with milkability traits, namely milking speed measured on a subjective scale [13,14]. These milking speed markers explain a fraction of the genetic variance associated with milk flow traits. It is likely that there are more genomic regions associated with milkability traits that have not been unveiled and some of these include genes, regulatory regions or some other sequence of DNA that behaves differently due to a single mutation causing a change in biological mechanisms associated with milk flow. Implementation of a GWAS using a dense 50k marker panel will aid in the discovery of unidentified QTL regions for milking speed traits. Significant markers could potentially represent causative mutations within previously identified genes, functional RNAs or regulatory regions, or more likely are not causative by themselves but in sufficient linkage disequilibrium (LD) to pick up the effect of the nearby causative factor. Identification of candidate gene regions could lead to a better understanding of the biological mechanisms that control milking flow in dairy cattle. Furthermore, differences in the number of identified regions and size of effects for individual SNPs can shed some light on the genetic architecture of milk flow related traits. The objective of this study was to identify SNP markers associated with milk flow traits including total milking time (TMT), ascending time (AT), time of plateau (TP), descending time (DT), maximum milk flow (MMF) and average milk flow (AVGF) by GWAS. As a result chromosomal regions would be identified that could include QTLs for milkability traits that can potentially improve selection based on genomic predictions.

## Results and discussion

### Significant SNPs

Among the milk flow traits investigated in this study heritabilities ranged from 0.42 (MMF) to 0.02 (AT) and reliabilities ranged from 0.60 (MMF) to 0.38 (TP) (Table 1). There were also 6,929 (TP) to 14,585 (MMF) markers that were significantly different from 0 for each of the milk flow traits that were identified across all 29 *Bos taurus* autosomes (BTA).

**Table 1:** Summary of heritabilities and reliabilities of estimated breeding values

**Trait**		**Reliability of EBV**	
		**Mean**	**Std. Dev.**
**TMT (min)**	0.11 ± 0.009	0.55	0.202
**AT (min)**	0.02 ± 0.006	0.46	0.170
**TP (min)**	0.32 ± 0.016	0.59	0.214
**DT (min)**	0.05 ± 0.007	0.38	0.160
**MMF (kg/min)**	0.42 ± 0.016	0.60	0.217
**AVGF (kg/min)**	0.29 ± 0.014	0.58	0.211

*TMT* – Total Milking Time; *AT* – Ascending Time; *TP* – Time of Plateau; *DT* – Descending Time; *MMF* – Maximum Milk Flow; *AVGF* – Average Milk Flow; *EBV* – Estimated Breeding Value.

**Figure 1:**
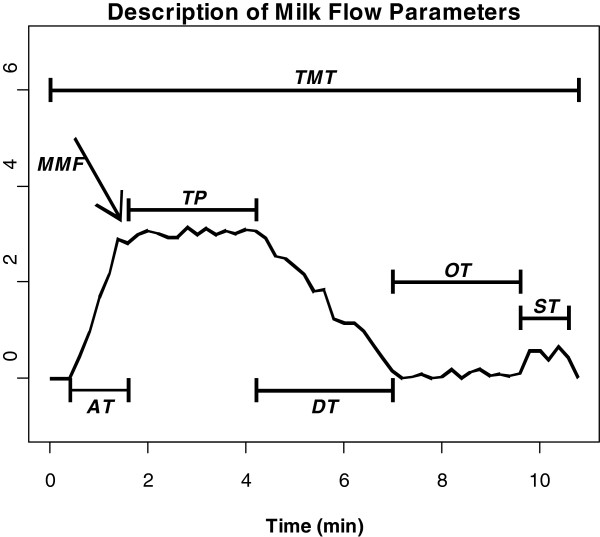
**Marker effects obtained for 35,044 SNPs for milk flow traits (Total milking time (min), Ascending Time (min), Time of Plateau (min), Descending time (min), Maximum milk flow (kg/min), Average milk flow (kg/min)) across all of the bovine autosomes.** Markers within MSPD QTL previously reported are labeled with “MSPD” and a purple point. The bold line signifies the upper and lower bound of the 95% confidence interval for each marker effect. Red and purple points signify markers with the 100 largest effects.

Markers across the genome with the largest absolute effects corresponding to setting a corrected p-value for multiple comparisons to P < 0.001, within each trait, were selected for further investigation and comparison, this effectively corresponded to approximately one hundred markers for each trait (112, 104, 108, 87, 71, 73, for MMF, TMT, AVGF, TP, AT, DT, respectively). To facilitate comparisons we arbitrarily selected markers with the largest 100 effects (Figure 1). These explained 6.3%, 5.1%, 5.3%, 6.4%, 6.2% and 5.4% of the total variance for TMT, AT, TP, DT, MMF and AVGF, respectively. Table 2 summarizes the combined effect of the 100 markers with largest effects for each trait. Many markers with the largest effects were either shared across traits or were within close proximity. Markers that were close (within 20 markers to the left or right) were considered to be within a single region. The largest region spanned 7.0 Mb. Additional file 1: Table S1 and Additional file 2: Table S2 identifies regions within QTL of interest, reporting the largest absolute effect within each region, emphasizing the 10 largest marker effects for each trait as well as names and locations of the SNP within the chromosome.

**Table 2:** Summary of markers with largest effects (p < 0.001) including the number of regions and number of chromosomes that they are found in

**Trait**^**1**^	**# of BTA**^**2**^	**# of regions**	**Combined absolute effect**^**3**^	**Combined relative variance**
TMT	28	77	38 s	6.3%
AT	27	79	2 s	5.1%
TP	28	78	9 s	5.3%
DT	27	76	43 s	6.4%
MMF	27	75	0.45 kg/min	6.2%
AVGF	28	77	0.26 kg/min	5.4%

^*1*^*TMT* – total milking time, *AT* – ascending time, *TP* – time of plateau, *DT* – descending time, *MMF* – maximum milk flow, *AVGF* – average milk flow.

^*2*^*BTA* – bos Taurus autosome.

^3^Combined Absolute Effect – refers to the absolute largest effect that the markers can have on each of the milk flow traits by summing absolute effects across each region.

Among all of the QTL previously mapped in the bovine genome only a few have been identified for milking speed (MSPD) measured on a subjective scale. There are a total of 14 MSPD QTL across 12 BTA identified among Holstein [14-16], Normande [16], Montbeliarde [16] and Finnish Ayrshire [17] dairy breeds. However, there have been no MSPD QTL reported for Brown Swiss. Nevertheless, significant markers identified from this study were located within all previously identified MSPD QTL within other breeds [14-17].

Udder morphometric traits (UT) (teat placement (TPL), udder attachment (UA), udder cleft (UC), udder composite index (UCI), udder depth (UDPTH), udder height (UHT) and udder width (UWDT)) are associated with milk flow traits [16]. It is understood that milk flow may be highly influenced by size, shape and overall confirmation of the udder [18]. Reduced milking performance has been recognized to be associated with larger or slacker teats as well and is influential in change of milking speed and time [19]. For this study all udder morphometric QTL were categorized as a single trait referred to as UT.

Other QTL potentially associated with milk flow are mammary health indicator traits (clinical mastitis (CM), somatic cell count (SCC) and somatic cell score (SCS)). Since most mammary infections are caused by bacterial invasion of the mammary gland through the teat canal cows with larger teat canals are more susceptible to mammary infections. Larger teat canals also increases MMF [20] and increases in MMF may result in an increased incidence of clinical mastitis. Both SCC and SCS have a positive genetic correlation with milk flow [3] and are considered to be indicator traits for clinical mastitis, therefore QTL for all three traits (SCC, SCS and CM) were considered in the analysis.

There is an unfavorable relationship between production traits (i.e. milk yield (MY) and 305 day lactation milk yield MY305) and milking time [3] thus QTL for these traits could possibly be co-located with significant markers for milk flow traits. Numerous milk yield QTL (MY, MY305, etc.) have been previously identified across the bovine genome in several breeds. Only MY QTL previously identified for Brown Swiss were here considered [21].

### Ascending time (AT)

There were 9,425 SNP marker effects that were significantly different from 0 (outside the 95% C.I.). Markers with the largest effect (n = 100) identified 79 distinct regions. Among these regions 41 were within QTL related to milk flow previously identified, including mastitis, udder and milk production QTL. Four of these regions located in BTA4 (70.7 to 70.9 Mb), BTA7 (52.9 to 56.5 Mb, 81.6 Mb) and BTA23 (21.3 to 23.7 Mb) were within previous MSPD QTL. The variance explained by these single regions was 0.123%, 0.06%, 0.05%, and 0.05% of the total genomic variance for AT, respectively. MSPD QTL that were previously identified in Holstein, French dairy cattle (Holstein, Normande and Montbeliarde) and Finnish Ayrshire mapped within BTA7 (35.9 – 106 Mb) [14], BTA23 (19.4-28.0 Mb) [17] and BTA4 (57.0 – 75.3 Mb) [14]. The largest marker effect (0.06 s) for AT (Hapmap38595-BTA-36915, 44.2 Mb) was located within a region on BTA15 (39.4 – 44.2 Mb) and explained 0.42% of the additive variance. UT (40.1 – 47.9 Mb) [22] and SCS (39.1 – 41.1 Mb) [23] QTL also mapped to this region on BTA15.

### Time at plateau (TP)

Time at plateau (TP) had the least number of effects that were significantly different from 0 (outside the 95% C.I.) at 6,929 markers across the entire genome. Among the 100 largest effects for TP there were 78 regions of which 41 were within QTL previously identified for traits associated with milking production traits. Two of these regions located in BTA6 (118.6 Mb) and BTA23 (35.5 to 38.2 Mb) were within MSPD QTL previously identified for French dairy cattle and Holstein on BTA6 (99.2 – 113 Mb) [16] and BTA23 (19.4-36.7 Mb) [17], respectively. The markers associated with MSPD QTL on BTA6 and BTA23 explained 0.06% and 0.21% of the genomic variance, respectively. The largest marker effect (0.18 s) was located on BTA25 (ARS-BFGL-NGS-114447, 40.8 Mb) explaining 0.12% of additive variance for TP. This marker mapped within SCS, UT (39.1 – 41.1 Mb) and MY (32.9 – 43.9 Mb) [14,24] QTL previously identified.

### Descending time (DT)

Descending time (DT) had 13,590 markers significantly different from 0 (outside the 95% C.I.). The 100 markers with largest effects identified 76 regions of which 36 were within QTL of interest. Descending time had the largest number of regions mapping within MSPD QTL. Three regions located in BTA7 (81.6 Mb, 93.1 to 93.3 Mb and 102.9 Mb) explaining a total of 0.23% of the genomic variance were within the MSPD QTL reported to be on BTA7 (35.9 – 106 Mb) [14] and another three regions located in BTA4 (70.7 to 70.9), BTA19 (52.3 Mb) and BTA23 (20.0 – 23.0) were within MSPD QTL explaining 0.11%, 0.06% and 0.05% of the total genomic variance, respectively. These QTL were previously identified on BTA4 (57.0 – 75.3 Mb) [14], BTA19 (51.5 – 53.5 Mb) [16] and BTA23 (19.4-36.7.0 Mb) [17]. The largest effect for DT (0.66 s) was the same marker that was largest for TMT, MMF and AVGF (Hapmap27408-BTA-143963, BTA6, 42.5 Mb) and explained 0.19% of the total genomic variance for DT. This marker was within 3 QTL identified in Holstein-Friesian including CM (26.7 – 65.9 Mb) [25] SCS (41.4 – 43.4 Mb) [26], and UT (34.6 – 44.2 Mb) [14].

### Total milking time (TMT)

Total milking time is essentially a combination of AT, TP and DT. There were 11,612 markers across all 29 BTA with predicted effects that were significantly different from 0 (outside the 95% C.I.) for TMT. The 100 largest markers effect were located in 77 distinct regions of which 37 with at least one marker within a previously identified QTL for traits associated with milk flow. Among these regions, there were 4 regions in BTA7 (52.9 to 56.5 Mb), BTA8 (55 Mb) and BTA23 (21.3 – 23.7 Mb and 35.5 to 38.2 Mb) within QTL previously identified for MSPD in Holstein, French dairy cattle and Finnish Ayrshire which explained 0.06%, 0.05%, 0.07% and 0.05% of the genomic variance for TMT, respectively. These QTL were also among other milking flow related QTL that were previously identified within BTA7 (35.9 – 106 Mb) [14], BTA8 (20.0 - 55.1 Mb) [16] and BTA23 (19.4-28.0 Mb) [17] including SCS, MY, CM and UT. Similar to DT the marker with the largest effect (0.84 s) which explained 0.21% of genomic variance for TMT was on BTA6 (Hapmap27408-BTA-143963, 42.5 Mb).

### Maximum milk flow (MMF)

Maximum milk flow (MMF) had the largest number of significant SNPs (14,585). There were 75 regions harboring the 100 largest markers for MMF. Of these regions 34 mapped within previously discovered QTL. Three of these regions on BTA4 (70.7 to 70.9), BTA7 (81.6 Mb) and BTA23 (21.3 to 23.7 Mb) explaining 0.12%, 0.04%, 0.07% of the genomic variance for MMF mapped within MSPD QTL on BTA4 (57.0 – 75.3 Mb) [14], BTA7 (35.9 – 106 Mb) [14] and BTA23 (19.4-36.7 Mb) [17]. The largest absolute effect (0.011 kg/min) was identified on BTA6 (Hapmap27408-BTA-143963, 42.5 Mb) and explained 0.22% of genomic variance for MMF similarly to TMT and DT.

### Average milk flow (AVGF)

Average milk flow (AVGF) had 14,178 significant markers across the entire genome. Among the 100 markers with largest effects there were 77 regions of which 33 within QTL of interest. Regions on BTA7 (81.6 Mb) and BTA23 (21.3 to 23.7 Mb) explained 0.05% and 0.06% of genomic variance for AVGF and were within two MSPD QTL (35.9 – 106 Mb) [17] (19.4-36.7 Mb) [17] on BTA7 and BTA23, respectively. The marker with the largest effect (0.006 kg/min) was also the same marker identified as having the largest effect for TMT, DT and MMF on BTA6 (Hapmap27408-BTA-143963, 42.5 Mb) and explained 0.23% of the genomic variance for AVGF.

### Common markers among milk flow traits

**Figure 2:**
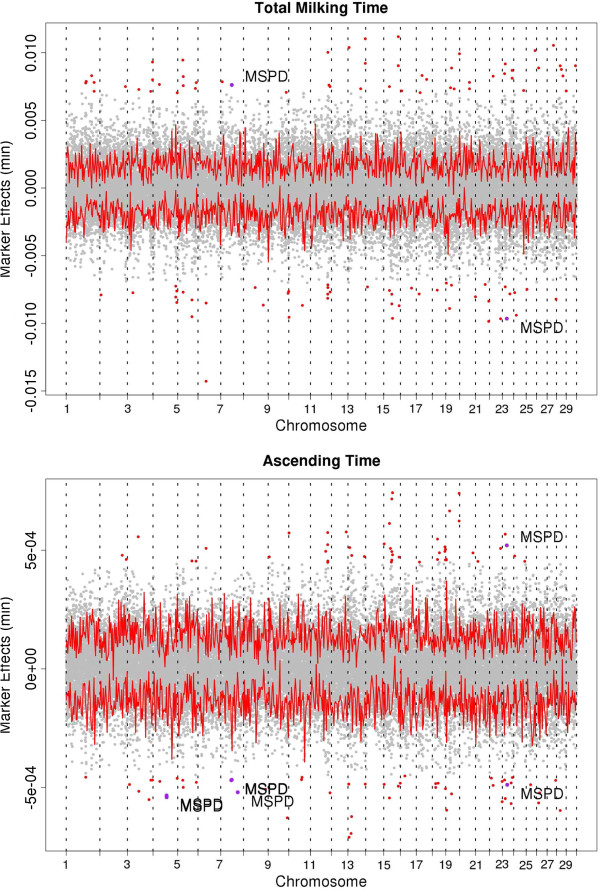
AT - Ascending Time; TP - Time of Plateau; DT - Descending Time; OT – Overmilking Time; ST - Stripping Time; TMT - Total Milking Time; MMF - Maximum Milk Flow.

As total milking time (TMT) is a composite measure of AT, TP and DT (Figure 2), it was expected that a large proportion of markers associated with TMT would also be included in the largest SNP effects for other traits. Among the regions harboring 100 of the largest SNP effects for TMT within QTL previously identified, only 4 regions were not shared with one of the other milking flow traits investigated in this study (Additional file 1: Table S1). Similarly AVGF had one region within a QTL that was not shared with any of the other traits and MMF did not have any unique regions within QTL. The overlap of AVGF was expected due to its strong genetic correlation with MMF, TP and TMT [3]. However, there were unique regions that were not within previously identified QTL.

As mentioned above the largest effect for TMT, DT, MMF and AVGF was a single marker (Hapmap27408-BTA-143963, 42.5 Mb) on BTA6 which was within a QTL rich region of 3 QTL for CM (26.7 – 65.9 Mb) [25] SCS (41.4 – 43.4 Mb) [26], and UT (34.6 – 44.2 Mb) [14]. Although this marker was not the largest effect for AT and TP it was within the top 40 largest effects. Further investigation within a 5 Mb region on each side of this marker revealed no annotated genes with obvious connection to milk flow.

While there were several common SNPs identified among the 100 with largest effect for each of the traits, additional unique SNPs were identified for each of the milk flow traits with TP having the most unique markers. This indicates that while milk flow traits show a medium to large genetic correlation [3] there might be regions of the genome that are uniquely associated with each trait.

A large number of markers identified for milk flow traits were in regions where QTL for MSPD, MY, SCC, SCS, UT and CM, were previously found (Additional file 1: Table S1), indicating that milking time is a complex trait that either incorporates many factors, influences several other traits or a combination of both. Although associations between milking speed, SCS, and clinical mastitis incidence has been somewhat controversial, it is generally accepted that fast milking whether measured subjectively [16,27] or electronically [28,29] is associated with higher levels of SCS. One possible explanation for this biological correlation could be the result of increased teat sphincter diameter [16]. This could result in an increase in milk flow and subsequently allow more pathogens to enter the mammary gland causing infection of the udder.

Finding markers within QTL associated with udder morphometric traits was expected. It has been reported that udder morphometric traits including increased udder size and teat size tend to negatively influence the efficiency of the milking machine therefore increasing the amount of time it takes to milk [18,30].

### Complexity of milk flow traits

**Figure 3:**
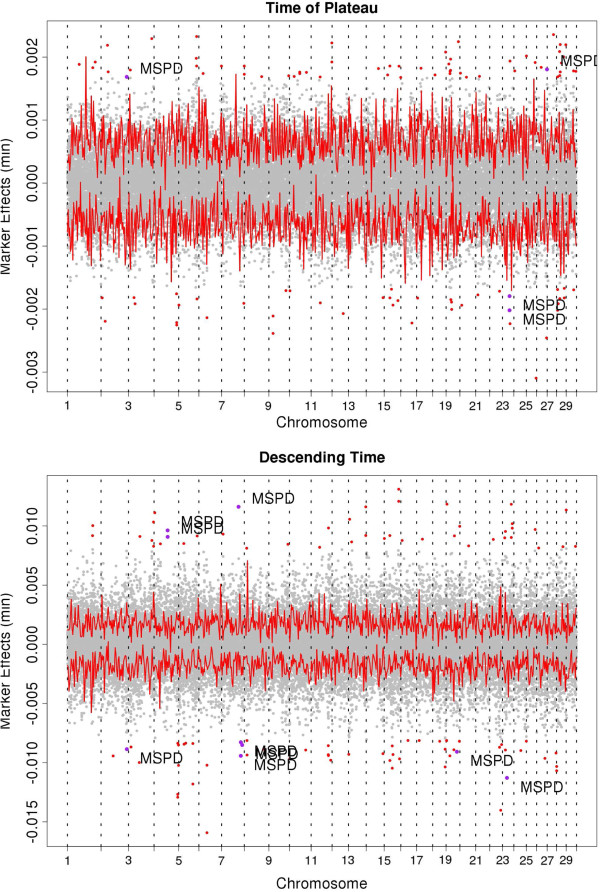
**Correlations between EBV and DGV.A**. Correlation between EBV and direct genomic value (DGV) predicted form all marker effects for TMT (total milking time), MMF (maximum milk flow), AVGF (average flow), AT (ascending time), TP (time at plateau), DT (descending time) **B**. Proportion of the correlation between EBV and DGV recovered (max correlation on panel **A**) from a finite number of markers (25, 50, 100, 300, 3000 and 10,000) with largest effects.

Only a handful of traits in dairy cattle, such as milk-fat composition are highly influenced by few genes [31,32]. In an effort to quantify the complexity of milk flow, Pearson’s correlations between predicted bull EBVs using a 6-trait animal model and the direct genomic values (DGV) obtained from all markers available for sires within the prediction set were computed (Figure 3A). These correlations ranged from 0.56 to 0.73. It is acknowledged that the EBVs were predicted from a multi-trait model while DGVs were predicted using single trait methods. Although these estimates may not be directly comparable they were used in the analysis given that they are the best predictor of the true breeding value of the data available for each of the methods of prediction.

Markers within each milk flow trait were sorted based on their absolute effect for the given trait of interest. Markers were isolated from the complete panel in subsets of 25, 50, 300, 3,000 and 10,000 SNPs with the largest effect. Genomic breeding values and genomic variances were then obtained from these subsets of markers.

Figure 3B depicts the proportion of the correlation coefficient depicted in 3A recovered by EBVs predicted from phenotypic data and DGVs obtained from subsets of markers with the largest effects for each of the milk flow traits, namely 25, 50, 300, 3,000 and 10,000 SNPs. In the figure correlation coefficient between EBVs and DGV predicted from 25 markers ranged from 0.13 to 0.33 which explains 19% to 46% of the correlation between EBV and DGV predicted using all of the markers, as illustrated in Figure 3A. There appeared to be two traits, AT and DT, for which panels of 25 markers captured twice as much correlation than, TMT, TP, MMF and AVGF. While it is possible that this may be the result of the markers capturing QTL with larger effects this is unlikely due to the low heritability that was estimated for these traits. The large proportion of the correlation explained within AT and DT is most likely the result of poor reliability for EBV obtained from phenotypic data. Poor reliable estimates of EBVs resulted in a lower overall correlation between the EBV and DGV (Figure 3A). Given that the correlation is lower in general it is possible that a smaller number of markers would explain a larger proportion of the correlation.

Among the other traits, with moderate heritabilities, breeding values obtained from 25 markers captured approximately one-fifth of the overall correlation. Increasing the number of markers from 25 to 3000 increased the correlation with approximately 0.45 for all traits. Correlations between EBVs and DGVs predicted from more than 3000 markers plateaued revealing a non-linear relationship between correlations estimated from differing number of markers, contrary to what would be expected under a pure polygenic architecture. This offers possible evidence for the presence of moderate size QTL, associated with milk flow.

Estimates of the additive genomic variance were computed from subsets of markers (25, 50, 100, 300, 3000, 10000, All). These variances were then compared to the genomic variance computed from all the markers and the proportion of the variance that can be explained by these subsets of markers were reported in Table 3. The proportion of the variance explained was similar across all milk flow traits (Table 3). Over half of the genomic variation was explained by 3,000 markers with the largest effects for each trait. When 10,000 markers are used approximately 84% of the genomic variance can be explained, roughly equivalent to the amount of genomic variance explained by all significant markers.

**Table 3:** Proportion of genomic variance that can be explained with different number of markers with largest effects

**# of markers**	**TMT**^**1**^	**AT**	**TP**	**DT**	**MMF**	**AVGF**
25	0.021	0.019	0.014	0.022	0.027	0.021
50	0.032	0.031	0.036	0.041	0.033	0.032
100	0.06	0.055	0.045	0.066	0.063	0.059
300	0.121	0.123	0.131	0.118	0.131	0.118
3000	0.500	0.501	0.502	0.506	0.520	0.522
10000	0.826	0.831	0.831	0.838	0.841	0.854
All	1	1	1	1	1	1

^*1*^*TMT* - Total Milking Time; *AT* - Ascending Time; *TP* - Time of Plateau; *DT* - Descending Time; *MMF* - Maximum Milk Flow; *AVGF* - Average Milk Flow.

There were a total of 9,844 markers (approximately 30% of overall number of SNPs) that were located within coding or regulatory regions. This proportion remained unchanged among significant markers with an average 3,553 markers that had a significant effect (Table 4). Similar results where obtained for subset of markers and no enrichment of coding SNPs was present among the most significant 100 markers While it appears that markers within genes and regulatory regions do not contribute more variation compared to an equal random set, density of markers for this analysis might not be sufficient to clearly separate the two components due to the large LD span in cattle.

**Table 4:** Number of markers within genes or regulatory region and the proportion of variation that can be explained by markers within genes

**Number of markers with largest effects**	**TMT**^**1**^	**AT**	**TP**	**DT**	**MMF**	**AVGF**
25	8	12	9	7	10	7
50	13	20	21	13	13	12
100	29	41	34	31	34	25
300	82	100	94	88	93	95
3000	940	929	917	935	939	929
10000	3,085	3,026	2,996	3,056	3,076	3,055
All significant markers	3,489	2,839	2,154	4,142	4,362	4,331
Total number of Markers within Gene or Regulatory Region	11,612	9,425	6,929	13,590	14,585	14,178
Proportion of Variation explained from Markers within genes and regulatory regions	31.4%	31.7%	30.7%	30.1%	29.9%	31.3%

^*1*^*TMT* - Total Milking Time^;^*AT* - Ascending Time; *TP* - Time of Plateau; *DT* - Descending Time; *MMF* - Maximum Milk Flow, *AVGF* – Average Milk Flow.

DGVs were predicted from significant marker effects within coding regions (DGV_gene_), and were compared to DGVs predicted from all significant markers (DGV_sig_) without respect to whether they were in a coding region or not. The median proportions DGV_sig_ that can be explained by DGV_gene_ were 0.35, 0.39, 0.73, 0.20, 0.43 and 0.34 for TMT, AT, TP, DT, MMF and AVGF, respectively. Time at plateau was the only trait where markers within coding regions explained a larger proportion of the breeding value than markers within non-coding regions. Significant markers for TP within genes had larger effects than the remaining significant markers suggesting that the set of markers used in this study may have in fact identified genes that influence TP.

### Functional processes associated to SNP affecting milk flow

Within the mammary gland, milk is initially secreted into small sacs called alveoli, from which it is ejected during milking [33]. Mammary alveoli are surrounded by smooth muscle (myoepithelial) cells [34], which are a prominent target cell for oxytocin [35]. Oxytocin stimulates contraction of myoepithelial cells, causing milk to be ejected into the ducts and cisterns [34]. Smooth muscle contractions in vertebrates are initiated by an increase in intracellular calcium [36]. In turns the intracellular calcium binds with calmodulin, which then binds and activates myosin light-chain kinase [36]. The calcium-calmodulin-myosin light-chain kinase complex phosphorylates myosin initiating contraction and activating the myosin ATPase [36]. By selecting the 1,000 markers with largest effects in AT, TP, DT, TMT, MMF and AVGF a total of 452 unique genes were identified using the R package *FunctSNP*[37]. Performing a functional annotation through DAVID [38], calmodulin-binding was the most significant functional category identified through ***sp_pir_keywords*** (nominal *P - Value* of 7.3E-3). A further search of the universal protein database [39] identified the ADCY (adenilate cyclase) gene (BTA4 76869665.76969459), a gene implicated in calmodulin induced events and calcium signaling pathway [40]. Furthermore, a significant KEGG pathway (nominal *P - Value* of 1.2E-2) was identified with 11 genes related to the calcium-signaling pathway (Additional file 3: Figure S1). Among these genes was the 5-hydroxytryptamine receptor 2A HTR2A, (BTA12 16823524.16889281) a serotonin receptor. Serotonin is a biogenic amine that is synthesized both in the enteric nervous system and the central nervous system [41] and the serotonergic system is involved in the regulation of oxytocin release [42]. Results the current GWAS are only suggestive and further investigation should be carried out to confirm or rule out our findings, but the current study identified potential areas for future studies targeted at particular pathways that include genes that may influence milk flow.

## Conclusions

In this study we have identified significant markers for six milk flow traits. There was an average of 11,720 markers significant for each of the milk flow traits assessed with a majority of markers having a small effect. These traits should be considered polygenic; however, we were able to collocate 10 regions with largest effects to seven QTL previously identified for milking speed. Each trait had significant markers that were in same region as MY and udder confirmation QTL. Phenotypic and genetic relationships between milking speed and mastitis, or its indicator traits, have been controversial, among the largest marker effects for each of the milk flow traits, there was an association with udder health QTL (SCC, SCS, or clinical mastitis) and milking speed QTL. Other significant regions for each of the traits were also identified but did not appear to be near any previously associated QTL or annotated gene requiring additional investigation to determine if these regions capture additional information that would be beneficial in understanding the biological mechanisms involved in milk flow.

Genetic improvement of milk flow using genomic tools has yet to be implemented. Whole genome selection methods should be considered as an alternative to using traditional selection methods or even considering the use of a subset of significant markers. This study verified that milk flow traits are complex and by including all information available from a SNP marker panel for all six milk flow traits in a selection index, milk flow pattern is taken into account on a genomic level, therefore it can be more advantageous in improving selection strategy. Incorporating all traits should decrease milk time while controlling for milk yield as well as control for clinical mastitis and its indicator traits of SCC and SCS and also other traits associated with udder size through indirect correlated response to selection in Italian Brown Swiss.

## Methods

Data for this study were provided by ANARB and included information spanning a 12 year period (1997 to 2008).

### Description of traits and animals

The dataset included 37,213 cows, daughters of 2,361 sires and 30,231 dams with pedigree information spanning seven generations. Milking release was measured once for each cow using a portable milk flow recorder (LactoCorder, WMB AG, Balgach, Switzerland). Milk flow characteristics were detected every 0.7 s and saved at intervals of 2.8 s. Milk flow was divided into six phases: 1) AT, period spanning milk flow greater than 0.5 kg/min until TP; 2) TP, period of steady milk flow; 3) DT, period from the end of TP until milk flow below 0.2 kg/min; 4) overmilking time, period between milk flow below 0.2 kg/min and group removal; 5) stripping time, period at end of milking, with milk flow greater than 0.2 kg/min and lasting for at least 4.2 s; and 6) overmilking time after stripping, period after stripping between a milk flow below 0.2 kg/min and the group removal after stripping time (Figure 2). The overall sum of all the periods corresponds to TMT. Additionally, MMF was recorded as the maximum flow preceding TP. The six traits investigated in this study were: TMT, AT, TP, DT, MMF and average milk flow (AVGF). A complete description of editing and collection procedures for these traits can be found in Gray et al. [3].

There were 1351 bulls genotyped from the Italian Brown Swiss population that had direct relationship ties with cows that have measurements of milk flow. Average progeny size for the bulls in the dataset was 28 ± 2.6 daughters with milk flow measurements available for analysis. Breeding values for the genotyped bulls were estimated using a multivariate 6-trait animal model similar to the model described in Gray et al. [3]. Breeding values were then de-regressed free from parental averages [43].

### SNP markers

Bulls were genotyped using the Illumina Bovine SNP50 BeadChip [9]. Markers with call rate < 0.90, minor allele frequency (MAF) < 0.05 and markers violating Hardy Weinberg equilibrium test, were all discarded from the analysis. After filtering, applying these quality criteria 33,074 SNPs on 29 bovine autosomes remained for analysis (Table 5).

**Table 5:** Number of markers per chromosome

**BTA**	**Number of markers**
1	2,184
2	1,784
3	1,659
4	1,603
5	1,310
6	1,476
7	1,477
8	1,597
9	1,335
10	1,405
11	1,363
12	1,066
13	1,150
14	1,168
15	1,083
16	1,027
17	1,042
18	871
19	898
20	1,029
21	906
22	833
23	738
24	807
25	671
26	685
27	607
28	628
29	672
Total	33,074

Markers within coding regions were flagged, in an effort to identify the proportion of significant markers that may target functional mutations within annotated genes.

We extracted coordinates for coding regions from the GFF3 file for *Bos taurus* assembly Btau 4.0 file [44] ( http://www.ensembl.org, Btau_4.0; April 2010), 500 kb upstream and downstream of each gene were taken as regulatory regions.

### Statistical analysis

#### SNP association tests

Shrinkage is often employed in QTL mapping when the number of markers to test is much larger than the individual observations [45]. Briefly shrinkage methods work by forcing some of the marker effects with trivial effects to 0. Different approaches for shrinking are available allowing fitting all markers simultaneously. Although not necessarily often these methods are implemented in a Bayesian version, which effectively, allows more flexibility in the strength, with which each marker is pushed toward 0. In the Bayesian-LASSO a double exponential prior is assigned to SNP variances [46]. Alternatively, inverted *χ*^2^ priors can be used [47]. Here we report on a GWAS for milk flow traits using regression through Bayesian LASSO.

The general model employed in the analysis had the form:

$$y_{\mathrm{ij}}=\mu +S_{i}+\beta x_{i}+e_{\mathrm{ij}}$$

Where y represents de-regressed breeding values, μ represents a mean; *S*_i_ is the random polygenic effect for the sires was fitted to prevent spurious associations due to population structure and had variance estimated from the data (**A**σ^2^_s_) with **A** representing the expected (based on pedigree) relationship matrix among the sires [8] with a SNP genotype of the i_th_ sire represented as *x*_*i*_ (−1, 0, and 1); β represents the regression coefficient for the SNP or the allele substitution effect and *e*_ij_ is the random residual (**I**σ^2^_e_) for each observation. A Gibbs sampling algorithm for all traits was implemented in R (R Development core Team 2009). For each analysis a single chain of 100,000 iterations was run with a burn-in period of 30,000 iterations. Although values for the λ shrinkage parameter can be sampled within each chain from their gamma prior distribution, in the final analysis we fixed the values of lambda based on the average of 5 shorter exploratory chains, which were run for each trait (50,000 iterations). Values of the λ parameters were similar across traits ranging from 21 for TMT, to 37 for AT.

For all analyses, thinning was applied and samples were stored every 30 iterations. Convergence of each chain was assessed both by visual inspection of the trace and the use of estimates of effective sample size for variances obtained through the *coda* package in R [48]. Inferences on the parameters were made from the mean of the posterior samples after burn-in.

An alternative method commonly referred to as Bayes Cπ [49] in which explicit model selection is employed and the proportion of markers with null effect is estimated from the data was employed to support the results obtained from the permutation method described above. Both methods attained similar results with an estimate of π (the proportion of null markers) ranging from 59% to 67% in the Bayes Cπ analysis in line with the reshuffling approach (data not shown).

#### Declaring significance

Shrinkage mapping is an efficient tool for whole genomic evaluations due to its scalability to large sets of markers, when compared to interval mapping LA [50] or LDLA analyses [51,52]. In several Bayesian shrinkage analyses no explicit model selection is performed and the equivalent result is obtained implicitly through the shrinkage process. As a result detection of QTL is performed by visual inspection of the resulting Manhattan plot. Formal methods of testing marker significance can also be used employing a posterior likelihood ratio test for the model with and without a particular marker included [53]. Alternatively non-parametric methods based on data shuffling can be employed. In the current study a permutation within chain was employed as proposed by Che and Xu [54]. Briefly, in their permutation strategy, at every *h* iteration of the current Markov chain the data is shuffled, where 1 <*h* <*L* with *L* being the length of the Markov Chain. For *h* =*L* permutation is equivalent to across chain permutation, while *h=*1 implies permutation in each iteration. The reshuffled chain then provides the 0.25 α × 100% and the (1–0.25 α) × 100% percentiles used as critical values for the analysis [54]. The within chain permutation is a strategy to obtain the posterior of the markers effect (regression coefficients) under the null model. While conceptually the method is no different from across chain permutation, the number of chains needed for the analysis is reduced to two. After an exploratory investigation, the permutation parameter h was set to 3 for the current analysis. Permutated marker effects were obtained from the posterior means of a 250,000 iteration chain.

#### Evaluation of markers for prediction

In an effort to evaluate the complexity of milk flow traits, the following approach was employed to determine the number of markers necessary to predict direct genomic values (DGV) for milk flow traits. Genotyped sires were partitioned based on their EBV reliabilities into a discovery set (EBV reliability > 0.60) and a prediction set (EBV reliability < 0.60) (Table 6). De-regressed breeding values for sires within the discovery set were utilized to obtain marker effects. Marker effects were then summed across all SNPs for each sire within the prediction data set, which is analogous to obtaining a breeding value that can be estimated using genotypic information alone. Since the true breeding value is unknown the best estimate for the true breeding value is the EBV and was used as the standard for comparison.

**Table 6:** Number of animals in discovery and prediction data set

	**Total milking time**	**Ascending time**	**Time of plateau**	Descending time^*^	**Maximum milk flow**	**Average milk flow**
Discovery^1^	653	260	748	760	780	720
Prediction^2^	630	1001	544	440	513	570

^1^ Reliablility > 0.60.

^2^ Reliablility < 0.60.

^*^For Desending Time the reliability threshold was changed from 0.60 to 0.50 to increase the number of animals available within the discovery set. Due to the low heritability associated with the trait an insufficient number of animals (n = 85) had reliabilities over 0.60.

Correlations were estimated between the EBV and DGV for each of the traits within the prediction set to demonstrate the association between breeding values obtained from marker effects and the best estimate of the true breeding values. There is a correlation between additive genetic variance σ^2^_g_ and genomic variance σ^2^_g_ with the latter proportional to the first. For the LASSO model employed in this investigation the relationship is as follow $\sigma_{g}^{2}=\frac{\sigma_{a}^{2}}{2\sum_{j=1}^{p}q_{j}\left(1-q\right)}$. In our analysis the posterior mean of each SNP effect was used to obtain genomic merit of individuals. Then the proportion of total genomic variance explained by subset of markers was obtained similarly to what proposed by Peters et al. [55]. Specifically, the overall genomic variance was obtained as the variance of the estimated DGVs, and the variance of subset of markers was obtained as the variance of DGVS calculated from the specific subset employed.

### Functional annotation

Functional annotation pathways search and the identification of potential candidate genes were performed through DAVID [38]. Identification of unique genes was perfomed on the bovine assembly Btau_4.0 ( http://www.ensembl.org, Btau_4.0; Nov 2011) using the R package *FunctSNP*[37].

## Competing interests

The authors declare that they have no competing interests.

## Authors’ contributions

KAG performed analysis and drafted the manuscript. CM and JPC designed the study. CM helped in the analyses and in drafting the manuscript, AR helped in editing the genomic information and helped revise the manuscript, AB, MD, ABS, JPC helped draft and revise the manuscript. All authors read and approved the final manuscript.

## Supplementary Material

Additional file 1 **Table S1.** Largest absolute marker effects for milk flow traits within QTL previously identified for bovin. Click here for file

Additional file 2 **Table S2.** Names of markers with largest effect in region for each trait. Click here for file

Additional file 3 **Figure S3.** The Calcium signaling pathway, stars represent associations identified in the current study. Click here for file
