# Supplementary material for: Estimates of marker effects for measures of milk flow in the Italian brown Swiss dairy cattle population
Source: BMC Vet Res. 2012 Oct 23;8:199. doi: 10.1186/1746-6148-8-199 (PMC3534398; doi:10.1186/1746-6148-8-199)
Supplement: Additional file 1 — Table S1. Largest absolute marker effects for milk flow traits within QTL previously identified for bovin. [file 1746-6148-8-199-S1.pdf]

Supplemental Table 1: Largest absolute marker effects for milk flow traits within QTL previously identified for bovine.

Chr	Region (MB)		Combined Marker Effect within each region						Previously
	Beg	End	TMT (s)	AT (s)	TP (s)	DT (s)	MMF (kg/min)	AVGF (kg/min)	identified QTL[1]
1	133.3	135.8	2.280		0.447				UT MY
2	4.0	6.8	2.154		0.715				UT
2	19.3	21.4			0.715				UT
2	29.0	30.3			0.538				UT
2	31.7	33.1			0.391				UT
2	55.9	59.0				2.673	0.023		UT
2	113.5	115.7		0.191					UT
2	128.9	134.2	5.022	0.162	0.846	6.613	0.076	0.036	UT MY
3	7.2	14.9		0.106	0.751	6.225	0.071		SCC
3	30.1	38.8	3.233		1.579			0.029	SCC
3	103.4	106.0		0.236					CM
3	110.6	113.4	4.334	0.203	0.792	4.832			CM MY
4	4.3	7.3				4.378	0.059		SCC
4	33.0	37.6	3.787	0.183		5.407		0.031	SCS
4	69.5	72.3		0.183		4.162	0.036		UT MSPD
4	115.0	118.4	3.794	0.191		5.323	0.054	0.044	SCC
5	25.8	29.1	2.950	0.270		3.179	0.041	0.022	UT
5	40.7	44.1				4.344			UT
5	89.7	93.9	4.715	0.188		5.685	0.067	0.036	UT
5	109.6	116.6	3.958	0.097	0.798	3.845	0.097	0.057	UT
5	119.4	120.6						0.021	UT
6	41.7	43.5	2.885	0.129	0.420	3.947	0.053	0.022	CM SCS UT
6	117.4	118.9			0.571				CM UT MSPD
7	52.6	57.6	2.933	0.237					SCS MSPD MY
7	80.0	83.1		0.231		5.082	0.050	0.023	SCC MSPD MY
7	91.7	95.3				5.627			MSPD
7	100.8	104.9				4.743			MSPD
8	11.9	16.4				4.015	0.048		SCS
8	53.8	55.9	2.156						CM SCC MSPD
8	79.9	82.9			0.635				CM SCC
9	90.0	92.1			0.708				CM UT
9	94.2	98.9	3.603	0.200		3.798	0.051	0.027	CM UT

[1] AT - Ascending Time; TP - Time of Plateau; DT - Descending Time; OT - Overmilking Time; ST - Stripping Time; TMT - Total Milking Time; MMF - Maximum Milk Flow

[2] Clinical Mastitis (CM), Milking Speed (MSPD), Milk Yield (MY), Somatic Cell Count (SCC), Somatic Cell Score (SCS), Udder morphometric QTL (UT)

Supplemental Table 1 continued

Chr	Region (MB)		Combined Marker Effect within each region						Previously
	Beg	End	TMT (s)	AT (s)	TP (s)	DT (s)	MMF (kg/min)	AVGF (kg/min)	identified QTL[1]
9	102.9	105	7.591		0.548		0.054	0.059	CM SCS UT
10	2.4	4.4			0.808				CM
10	20.6	21.8			0.362				UT
10	50.4	52.1			0.837				UT SCC
10	62.1	69.7	4.075	0.167				0.023	SCS UT SCC
10	82.0	86.2			0.523	4.997			SCS UT
11	37.8	41.7			0.808				SCC
11	85.2	88.1	7.950	0.407		8.648	0.088	0.049	SCC MY
12	53.8	55.5					0.039	0.021	UT
12	59.8	62.2			0.463				UT
13	2.0	4.5					0.067	0.039	UT
13	6.7	10.0		0.170					UT
13	14.5	19.5		0.293		5.427			SCS UT MY
13	63.3	66.0	3.321						MY SCS UT
13	78.8	80.3		0.198					UT
13	81.7	83.9	4.510	0.256		6.127	0.067	0.029	UT
14	53.4	55.4			0.517				SCS
15	22.1	23.1			0.260				CM
15	33.5	38.2	5.164	0.512	0.877	4.667	0.109	0.058	SCS
15	43.1	45.4	3.664	0.258	0.611	3.900	0.049	0.025	SCS UT
16	27.0	30.4		0.235					UT
17	54.1	56.8					0.039	0.021	UT MY
18	12.8	16.8		0.237			0.042	0.025	SCS
18	21.1	27.1	5.037	0.213					SCS UT
18	32.8	35.1		0.286			0.053	0.026	UT
18	58.7	65.4		0.277	0.717	4.537	0.070	0.038	SCS UT
19	15.6	16.6			0.440				UT
19	18.1	21.0	3.697	0.322	1.061	3.392	0.036	0.021	UT SCS
19	25.6	28.8	5.024	0.249	1.033		0.058		SCS UT
19	36.9	40.2				4.389			SCS
19	50.7	53.7				4.127			SCS
20	6.7	8.0			0.448				UT MSPD
20	40.0	42.3				1.983			UT

[1] AT - Ascending Time; TP - Time of Plateau; DT - Descending Time; OT - Overmilking Time; ST - Stripping Time; TMT - Total Milking Time; MMF - Maximum Milk Flow

[2] Clinical Mastitis (CM), Milking Speed (MSPD), Milk Yield (MY), Somatic Cell Count (SCC), Somatic Cell Score (SCS), Udder morphometric QTL (UT)

Supplemental Table 1 continued

Chr	Region (MB)		Combined Marker Effect within each region						Previously
	Beg	End	TMT (s)	AT (s)	TP (s)	DT (s)	MMF (kg/min)	AVGF (kg/min)	identified QTL[1]
20	45.1	47.2	4.889						UT
20	60.6	62.8		0.180					UT MY
21	11.6	14.7			0.417				CM SCS SCC
21	19.3	21.3			0.773				CM UT
21	48.5	51.3	1.967				0.052		SCS
22	7.9	10.3		0.194					UT
23	10.5	13.1	3.535	0.213		4.327	0.053	0.025	SCS UT
23	13.9	15.6	4.736	0.319		4.036			UT
23	20.0	23.0	2.554	0.144		2.885	0.036	0.019	UT MSPD
23	36.6	39.4	4.751		0.882				SCS UT MSPD
23	46.9	48.9		0.312					SCC
24	33.9	35.9				4.115			SCS UT
24	47.3	50.0	2.764						UT
24	54.6	56.9		0.151					UT
25	17.1	19.8		0.223					SCS
25	21.1	23.4						0.019	UT
25	35.9	38.2	4.200		0.769	3.884	0.040	0.029	MY SCS
25	40.0	41.8			0.808				MY SCS UT
26	9.9	12.4	3.234	0.213		3.182	0.034	0.020	SCS UT
26	14.6	17.4		0.213					SCS UT
26	22.4	25.2			0.609				MY UT
26	40.1	43.1				5.952	0.065		CM SCS UT SCC
27	31.0	33.4	4.974		0.714			0.031	CM UT
28	3	7.0			0.865			0.021	UT
28	16.3	18.7	4.873	0.206	1.002			0.036	UT
28	22.2	25.4	3.505		0.507		0.052	0.023	UT
29	46.2	48.3	3.610		0.186	4.186	0.056	0.028	UT SCS

[1] AT - Ascending Time; TP - Time of Plateau; DT - Descending Time; OT - Overmilking Time; ST - Stripping Time; TMT - Total Milking Time; MMF - Maximum Milk Flow

[2] Clinical Mastitis (CM), Milking Speed (MSPD), Milk Yield (MY), Somatic Cell Count (SCC), Somatic Cell Score (SCS), Udder morphometric QTL (UT)
