# Supplementary material for: Estimates of marker effects for measures of milk flow in the Italian brown Swiss dairy cattle population
Source: BMC Vet Res. 2012 Oct 23;8:199. doi: 10.1186/1746-6148-8-199 (PMC3534398; doi:10.1186/1746-6148-8-199)
Supplement: Additional file 2 — Table S2. Names of markers with largest effect in region for each trait. [file 1746-6148-8-199-S2.pdf]

Supplemental Table 2 : Names of markers with largest effect in region for each trait

Chr	Region		Name of Marker with Largest Effect in each region [1]					
	Beg	End	TMT (s)	AT (s)	TP (s)	DT (s)	MMF (kg/min)	AVGF (kg/min)
1	133.3	135.8	ARS-BFGL-NGS-108336		ARS-BFGL-NGS-108336			
2	4	6.8	ARS-BFGL-NGS-24537		ARS-BFGL-NGS-24537			
2	19.3	21.4			BTA-46612-no-rs			
2	29	30.3			Hapmap61002-rs29016992			
2	31.7	33.1			BTA-104779-no-rs			
2	55.9	59				BTB-00099725	BTB-00099725	
2	113.5	115.7		ARS-BFGL-BAC-34939				
2	128.9	134.2	ARS-BFGL-NGS-33709	ARS-BFGL-NGS-113511	ARS-BFGL-NGS-108175	ARS-BFGL-NGS-2286	ARS-BFGL-NGS-2286	ARS-BFGL-NGS-108175
3	7.2	14.9		ARS-BFGL-NGS-33910	ARS-BFGL-NGS-33910			

[1] AT - Ascending Time; TP - Time of Plateau; DT - Descending Time; OT - Overmilking Time; ST - Stripping Time; TMT - Total Milking Time; MMF - Maximum Milk Flow

Supplemental Table 2 : Continued

Chr	Region		Name of Marker with Largest Effect in each region [1]					
	Beg	End	TMT (s)	AT (s)	TP (s)	DT (s)	MMF (kg/min)	AVGF (kg/min)
3	30.1	38.8	BTA-67383-no-rs		Hapmap55463-rs29026005			BTA-67383-no-rs
3	103.4	106		ARS_BFGL-NGS-1038				
3	110.6	113.4	ARS-BFGL-NGS-74948	ARS-BFGL-NGS-110683	ARS-BFGL-NGS-74948	ARS-BFGL-NGS-110683		
4	4.3	7.3				ARS-BFGL-NGS-104086	ARS-BFGL-NGS-104086	
4	33	37.6	BTA-97585-no-rs	Hapmap33884-BES3_Contig263_765		BTA-97585-no-rs		BTB-01793118
4	69.5	72.3		ARS-BFGL-NGS-102713		ARS-BFGL-NGS-36876	ARS-BFGL-NGS-36876	
4	115	118.4				ARS-BFGL-NGS-27061	Hapmap54131-rs29019697	Hapmap54131-rs29019697
5	25.8	29.1	ARS-BFGL-NGS-7725	Hapmap39353-BTA-73120		ARS-BFGL-NGS-7725	ARS-BFGL-NGS-7725	ARS-BFGL-NGS-7725
5	40.7	44.1						

[1] AT - Ascending Time; TP - Time of Plateau; DT - Descending Time; OT - Overmilking Time; ST - Stripping Time; TMT - Total Milking Time; MMF - Maximum Milk Flow

Supplemental Table 2 : Continued

Chr	Region		Name of Marker with Largest Effect in each region [1]					
	Beg	End	TMT (s)	AT (s)	TP (s)	DT (s)	MMF (kg/min)	AVGF (kg/min)
5	89.7	93.9	BTA-111858-no-rs	BTA-111858-no-rs		BTA-111858-no-rs	BTA-111858-no-rs	BTA-111858-no-rs
5	109.6	116.6	ARS-BFGL-NGS-13748		ARS-BFGL-NGS-13748		ARS-BFGL-NGS-44354	ARS-BFGL-NGS-13748
5	119.4	120.6						ARS-BFGL-NGS-110517
6	41.7	43.5	Hapmap27408-BTA-143963	Hapmap27408-BTA-143963	Hapmap27408-BTA-143963	Hapmap27408-BTA-143963	Hapmap27408-BTA-143963	Hapmap27408-BTA-143963
6	117.4	118.9			ARS-BFGL-NGS-72630			
7	52.6	57.6	ARS-BFGL-NGS-62351	Hapmap50471-BTA-78923				
7	80	83.1		ARS-BFGL-NGS-114593		ARS-BFGL-NGS-114593	ARS-BFGL-NGS-114593	ARS-BFGL-NGS-114593
7	91.7	95.3						

[1] AT - Ascending Time; TP - Time of Plateau; DT - Descending Time; OT - Overmilking Time; ST - Stripping Time; TMT - Total Milking Time; MMF - Maximum Milk Flow

Supplemental Table 2 : Continued

Chr	Region		Name of Marker with Largest Effect in each region [1]					
	Beg	End	TMT (s)	AT (s)	TP (s)	DT (s)	MMF (kg/min)	AVGF (kg/min)
7	100.8	104.9				Hapmap53461-rs29027660		
8	11.9	16.4				ARS-BFGL-NGS-50811	ARS-BFGL-NGS-26808	
8	53.8	55.9	Hapmap59304-rs29025230					
8	79.9	82.9			BTA-110160-no-rs			
9	90	92.1			BTB-01839335			
9	94.2	98.9	ARS-BFGL-NGS-55179	ARS-BFGL-NGS-9633		ARS-BFGL-NGS-9633	ARS-BFGL-NGS-9633	ARS-BFGL-NGS-55179
9	102.9	105	ARS-BFGL-NGS-114800		ARS-BFGL-NGS-117605		ARS-BFGL-NGS-26776	ARS-BFGL-NGS-26776
10	2.4	4.4			Hapmap59914-rs29012068			
10	20.6	21.8			ARS-BFGL-NGS-28483			

[1] AT - Ascending Time; TP - Time of Plateau; DT - Descending Time; OT - Overmilking Time; ST - Stripping Time; TMT - Total Milking Time; MMF - Maximum Milk Flow

Supplemental Table 2 : Continued

Chr	Region		Name of Marker with Largest Effect in each region [1]					
	Beg	End	TMT (s)	AT (s)	TP (s)	DT (s)	MMF (kg/min)	AVGF (kg/min)
10	50.4	52.1			BTB-00427715			
10	62.1	69.7	BTA-98729-no-rs	Hapmap59063-rs29016349				BTA-98729-no-rs
10	82	86.2			BTB-00441709	ARS-BFGL-NGS-112698		
11	37.8	41.7			ARS-BFGL-NGS-114070			
11	85.2	88.1	ARS-BFGL-NGS-116951	ARS-BFGL-NGS-116253		ARS-BFGL-NGS-116951	ARS-BFGL-NGS-35730	ARS-BFGL-NGS-116253
12	53.8	55.5					ARS-BFGL-NGS-26583	ARS-BFGL-NGS-26583
12	59.8	62.2			BTB-01011791			
13	2	4.5	BTB-01221050	BTB-01221050		BTB-01221050	BTB-01221050	BTB-01221050
13	6.7	10		ARS-BFGL-NGS-93056				

[1] AT - Ascending Time; TP - Time of Plateau; DT - Descending Time; OT - Overmilking Time; ST - Stripping Time; TMT - Total Milking Time; MMF - Maximum Milk Flow

Supplemental Table 2 : Continued

Chr	Region		Name of Marker with Largest Effect in each region [1]					
	Beg	End	TMT (s)	AT (s)	TP (s)	DT (s)	MMF (kg/min)	AVGF (kg/min)
13	14.5	19.5		ARS-BFGL-NGS-35631		ARS-BFGL-NGS-114762		
13	63.3	66	ARS-BFGL-NGS-113964					
13	78.8	80.3		ARS-BFGL-NGS-112395				
13	81.7	83.9	ARS-BFGL-NGS-39164	ARS-BFGL-NGS-39164		ARS-BFGL-NGS-39164	ARS-BFGL-NGS-39164	ARS-BFGL-NGS-39164
14	53.4	55.4			BTA-90428-no-rs			
15	22.1	23.1			ARS-BFGL-NGS-85535			
15	33.5	38.2		ARS-BFGL-NGS-2713	BTA-08811-rs29022367		ARS-BFGL-NGS-2713	ARS-BFGL-NGS-2713
15	43.1	45.4	Hapmap38595-BTA-36915	Hapmap38595-BTA-36915	Hapmap38595-BTA-36915	Hapmap38595-BTA-36915	Hapmap38595-BTA-36915	Hapmap38595-BTA-36915
16	27	30.4		ARS-BFGL-BAC-33348				

[1] AT - Ascending Time; TP - Time of Plateau; DT - Descending Time; OT - Overmilking Time; ST - Stripping Time; TMT - Total Milking Time; MMF - Maximum Milk Flow

Supplemental Table 2 : Continued

Chr	Region		Name of Marker with Largest Effect in each region [1]					
	Beg	End	TMT (s)	AT (s)	TP (s)	DT (s)	MMF (kg/min)	AVGF (kg/min)
17	54.1	56.8					Hapmap54286-ss46526589	Hapmap54286-ss46526589
18	12.8	16.8		ARS-BFGL-NGS-24837	ARS-BFGL-NGS-29564		ARS-BFGL-NGS-24837	ARS-BFGL-NGS-24837
18	21.1	27.1	ARS-BFGL-NGS-28677	ARS-BFGL-NGS-2512				
18	32.8	35.1		ARS-BFGL-NGS-22634			Hapmap23714-BTA-43016	
18	58.7	65.4		ARS-BFGL-NGS-49873	ARS-BFGL-NGS-38620	Hapmap40847-BTA-98475	ARS-BFGL-NGS-38620	ARS-BFGL-NGS-38620
19	15.6	16.6			ARS-BFGL-NGS-35416			
19	18.1	21	Hapmap40170-BTA-20573	Hapmap40170-BTA-20573	ARS-BFGL-NGS-20809	Hapmap40170-BTA-20573	Hapmap40170-BTA-20573	Hapmap40170-BTA-20573
19	25.6	28.8	BTB-00745347	BTB-00745347	ARS-BFGL-NGS-114201		BTB-00745347	
19	36.9	40.2				Hapmap49617-BTA-45355		

[1] AT - Ascending Time; TP - Time of Plateau; DT - Descending Time; OT - Overmilking Time; ST - Stripping Time; TMT - Total Milking Time; MMF - Maximum Milk Flow

Supplemental Table 2 : Continued

Chr	Region		Name of Marker with Largest Effect in each region [1]					
	Beg	End	TMT (s)	AT (s)	TP (s)	DT (s)	MMF (kg/min)	AVGF (kg/min)
19	50.7	53.7				Hapmap39750-BTA-45775		
20	6.7	8			ARS-BFGL-NGS-98789			
20	40	42.3				BTB-00783355		
20	45.1	47.2	BTB-00786048					
20	60.6	62.8		BTB-00794279				
21	11.6	14.7		Hapmap30803-BTA-135661	ARS-BFGL-NGS-58825			
21	19.3	21.3			ARS-BFGL-NGS-16989			
21	48.5	51.3	Hapmap52435-rs29012295				ARS-BFGL-NGS-2684	
22	7.9	10.3		ARS-BFGL-NGS-119364				
23	10.5	13.1	Hapmap26666-BTA-137044	Hapmap26666-BTA-137044		Hapmap26666-BTA-137044	Hapmap26666-BTA-137044	Hapmap26666-BTA-137044

[1] AT - Ascending Time; TP - Time of Plateau; DT - Descending Time; OT - Overmilking Time; ST - Stripping Time; TMT - Total Milking Time; MMF - Maximum Milk Flow

Supplemental Table 2 : Continued

Chr	Region		Name of Marker with Largest Effect in each region [1]					
	Beg	End	TMT (s)	AT (s)	TP (s)	DT (s)	MMF (kg/min)	AVGF (kg/min)
23	13.9	15.6	ARS-BFGL-NGS-17887	ARS-BFGL-NGS-42298		ARS-BFGL-NGS-17887		
23	20	23	ARS-BFGL-NGS-25108	ARS-BFGL-NGS-25108		ARS-BFGL-NGS-25108	ARS-BFGL-NGS-25108	ARS-BFGL-NGS-25108
23	36.6	39.4	ARS-BFGL-NGS-115866		ARS-BFGL-NGS-115866			
23	46.9	48.9		Hapmap53926-rs29025235				
24	33.9	35.9				BTB-00886759		
24	47.3	50	ARS-BFGL-NGS-24167					
24	54.6	56.9		Hapmap51262-BTA-58394				
25	17.1	19.8		ARS-BFGL-NGS-3834				
25	21.1	23.4						Hapmap50414-BTA-59706

[1] AT - Ascending Time; TP - Time of Plateau; DT - Descending Time; OT - Overmilking Time; ST - Stripping Time; TMT - Total Milking Time; MMF - Maximum Milk Flow

Supplemental Table 2 : Continued

Chr	Region		Name of Marker with Largest Effect in each region [1]					
	Beg	End	TMT (s)	AT (s)	TP (s)	DT (s)	MMF (kg/min)	AVGF (kg/min)
25	35.9	38.2	ARS-BFGL-BAC-40619		ARS-BFGL-BAC-40619	ARS-BFGL-BAC-40619	ARS-BFGL-BAC-40619	ARS-BFGL-BAC-40619
25	40	41.8			ARS-BFGL-NGS-114447			
26	9.9	12.4	BTB-01667023	BTB-01667023		BTB-01667023	BTB-01667023	BTB-01667023
26	14.6	17.4		ARS-BFGL-NGS-4442				
26	22.4	25.2			Hapmap38196-BTA-114696			
26	40.1	43.1				DPI-31	DPI-31	
27	31	33.4	Hapmap42770-BTA-103418		Hapmap42770-BTA-103418			Hapmap42770-BTA-103418
28	3	7			Hapmap57035-rs29025683			Hapmap53582-rs29012732

[1] AT - Ascending Time; TP - Time of Plateau; DT - Descending Time; OT - Overmilking Time; ST - Stripping Time; TMT - Total Milking Time; MMF - Maximum Milk Flow

Supplemental Table 2 : Continued

Chr	Region		Name of Marker with Largest Effect in each region [1]					
	Beg	End	TMT (s)	AT (s)	TP (s)	DT (s)	MMF (kg/min)	AVGF (kg/min)
28	16.3	18.7	ARS-BFGL-NGS-1363	ARS-BFGL-NGS-1363	ARS-BFGL-NGS-1363			ARS-BFGL-NGS-1363
28	22.2	25.4	BTB-01462216		BTB-01462216		BTB-01462216	BTB-01462216
29	46.2	48.3	ARS-BFGL-NGS-70165			ARS-BFGL-NGS-70165	ARS-BFGL-NGS-70165	ARS-BFGL-NGS-111472

[1] AT - Ascending Time; TP - Time of Plateau; DT - Descending Time; OT - Overmilking Time; ST - Stripping Time; TMT - Total Milking Time; MMF - Maximum Milk Flow
