# Supplementary material for: Estimates of marker effects for measures of milk flow in the Italian brown Swiss dairy cattle population
Source: BMC Vet Res. 2012 Oct 23;8:199. doi: 10.1186/1746-6148-8-199 (PMC3534398; doi:10.1186/1746-6148-8-199)

**Supplemental Figure 1**: The Calcium signaling pathway, stars represent associations identified in the current study
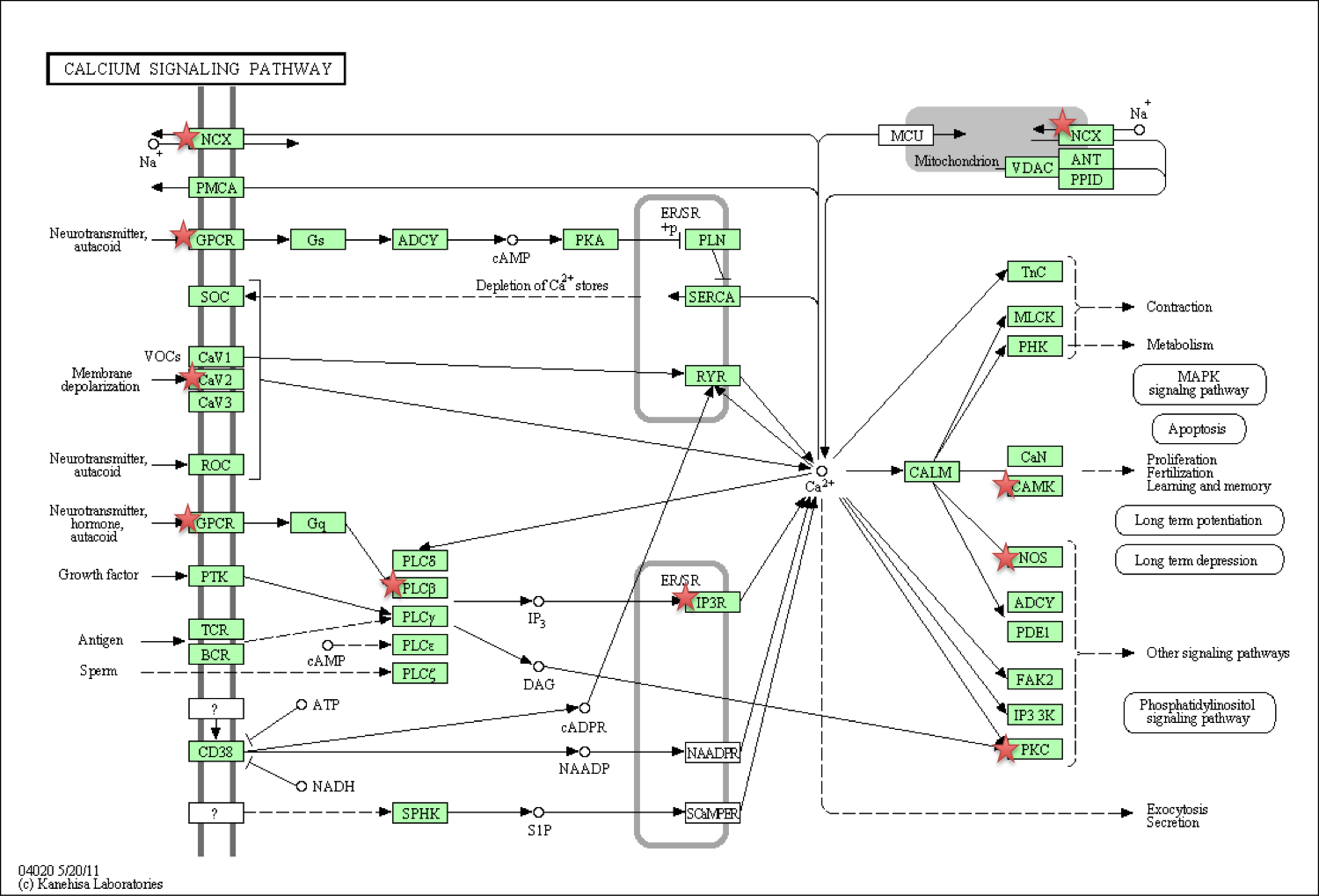

Supplement: Additional file 3 — Figure S3. The Calcium signaling pathway, stars represent associations identified in the current study. [file 1746-6148-8-199-S3.docx]
